# Reproducibility of left atrial function using cardiac magnetic resonance imaging

**DOI:** 10.1007/s00330-020-07399-z

**Published:** 2020-10-30

**Authors:** Aseel Alfuhied, Benjamin A. Marrow, Sara Elfawal, Gaurav S. Gulsin, Mathew P. Graham-Brown, Christopher D. Steadman, Prathap Kanagala, Gerry P. McCann, Anvesha Singh

**Affiliations:** 1 Department of Cardiovascular Sciences, Cardiovascular Theme National Institute for Health Research (NIHR) Leicester Biomedical Research Centre, Glenfield Hospital, University of Leicester, Groby Road, Leicester, LE3 9QP UK; 2 King Saud bin Abdulaziz University for Health Sciences, Riyadh, Kingdom of Saudi Arabia; 3 Department of Radiology, University Hospitals of Leicester NHS Trust, Leicester, UK; 4 John Walls Renal Unit, University Hospitals Leicester NHS Trust, Leicester, UK; 5 Department of Cardiology, Poole Hospital NHS Foundation Trust, Poole, UK

**Keywords:** Left atrial function, Cardiovascular diseases, Magnetic resonance imaging, Reproducibility of results

## Abstract

**Objectives:**

To determine the test-retest reproducibility and observer variability of CMR-derived LA function, using (i) LA strain (LAS) and strain rate (LASR), and (ii) LA volumes (LAV) and emptying fraction (LAEF).

**Methods:**

Sixty participants with and without cardiovascular disease (aortic stenosis (AS) (*n* = 16), type 2 diabetes (T2D) (*n* = 28), end-stage renal disease on haemodialysis (*n* = 10) and healthy volunteers (*n* = 6)) underwent two separate CMR scans 7–14 days apart. LAS and LASR, corresponding to LA reservoir, conduit and contractile booster-pump function, were assessed using Feature Tracking software (QStrain v2.0). LAEF was calculated using the biplane area length method (QMass v8.1). Both were assessed using 4- and 2-chamber long-axis standard steady-state free precession cine images, and average values were calculated. Intra- and inter-observer variabilities were assessed in 10 randomly selected participants.

**Results:**

The test-retest reproducibility was moderate to poor for all strain and strain rate parameters. Overall, strain and strain rate corresponding to reservoir phase (LAS_r, LASR_r) were the most reproducible, yielding the smallest coefficient of variance (CoV) (29.9% for LAS_r, 28.9% for LASR_r). The test-retest reproducibility for LAVs and LAEF was good: LAVmax CoV = 19.6% ICC = 0.89, LAVmin CoV = 27.0% ICC = 0.89 and total LAEF CoV = 15.6% ICC = 0.78. The inter- and intra-observer variabilities were good for all parameters except for conduit function.

**Conclusion:**

The test-retest reproducibility of LA strain and strain rate assessment by CMR utilising Feature Tracking is moderate to poor across disease states, whereas LA volume and emptying fraction are more reproducible on CMR. Further improvements in LA strain quantification are needed before widespread clinical application.

**Key Points:**

• *LA strain and strain rate assessment using Feature Tracking on CMR has moderate to poor test-retest reproducibility across disease states.*

• *The test-retest reproducibility for the biplane method of assessing LA function is better than strain assessment, with lower coefficient of variances and narrower limits of agreement on Bland-Altman plots.*

• *Biplane LA volumetric measurement also has better intra- and inter-observer variability compared to strain assessment.*

**Electronic supplementary material:**

The online version of this article (10.1007/s00330-020-07399-z) contains supplementary material, which is available to authorized users.

## Introduction

Left atrial (LA) remodeling is associated with left ventricular (LV) diastolic dysfunction [1]. LA volume (LAV) and emptying fraction (LAEF) are recognised as predictors of adverse outcomes across a range of cardiovascular diseases associated with LV diastolic dysfunction [2, 3], including aortic stenosis (AS) [4], type 2 diabetes (T2D) [5], chronic kidney disease [6], and heart failure with preserved and reduced ejection fraction [7, 8]. In addition, LA remodeling post-intervention such as aortic valve replacement predicts long-term outcome [9].

LA strain is an emerging imaging marker of LA function, which describes LA deformation. Typically, LA function is assessed by traditional measures such as LAV and LAEF. Assessing LA deformation may overcome the limitations of volumetric assessment such as geometric assumptions using the biplane area length method, and may also provide very early detection of functional impairment [10]. Strain abnormalities, even in the presence of normal LA size, have previously been shown in diabetic and hypertensive patients [10] and patients with hypertrophic cardiomyopathy [11]. LA strain has been reported to correlate with LV filling pressure [12–14] and is a sensitive marker detecting early LV diastolic dysfunction [14]. It is also recognised as a predictor of adverse cardiovascular outcomes in women in the general population [15] and in diseases that are associated with ventricular diastolic dysfunction [16–19].

Strain and strain rate measurement is now possible from routinely acquired cine images. This may be advantageous because of the unlimited imaging windows provided by CMR which is recognised as the gold standard technique for quantification of ventricular volumes and for myocardial tissue characterisation. However, the test-retest reproducibility of CMR-derived LA strain has not yet been reported in any patient groups. Good test-retest reproducibility is vital when monitoring treatment effect or disease progression in longitudinal studies. We aimed to determine the test-retest reproducibility and inter/intra-observer variability of CMR-derived LA function, using (i) LA strain (LAS) and strain rate (LASR), and (ii) LAV and LAEF using the biplane area length method in a range of subjects with and without cardiovascular disease.

## Methodology

### Population

Sixty participants were included: AS (*n* = 16), T2D (*n* = 28), end-stage renal disease on haemodialysis (*n* = 10) and healthy volunteers (*n* = 6). Participants were prospectively recruited for ethically approved studies at a single tertiary cardiac centre. Inclusion and exclusion criteria were as previously published: AS [20, 21], T2D [22, 23], and haemodialysis [24]. For the AS cohort, transthoracic echocardiography was performed on the same day to evaluate AS severity [25]. The healthy subjects [23] were non-diabetic, devoid of known cardiovascular disease and with normal electrocardiography (ECG), cardiopulmonary exercise testing, echocardiography and CMR.

### CMR imaging

All subjects underwent two CMR scans 7–14 days apart, on the same scanner for each participant, using standardised protocols. CMR scans for patients with AS (*n* = 10), T2D (*n* = 17), haemodialysis (*n* = 10) and healthy volunteers (*n* = 6) were conducted on a 3-Tesla scanner (Siemens Skyra). The rest of the subjects (*n* = 17) were scanned on a 1.5-Tesla scanner (Siemens Avanto or Siemens Aera). Haemodialysis patients were scanned on their non-dialysis day and not after their long break to standardise their volume status as far as possible. All subjects were in sinus rhythm and scanned using retrospective ECG gating. Long-axis (2-, 3- and 4-chamber) cine images were acquired before contrast administration in all subjects, using a steady-state free precession end-expiratory breath-hold sequence (typical parameters: slice thickness of 8 mm, matrix 256 × 204, field of view variable 300–360 × 360–420, TR 45 ms, TE 1.2 ms, flip angle 45°). Short-axis LV cine stacks were acquired to enable derivation of LV volumes, mass and function.

### CMR analysis

Image analysis was performed offline using dedicated software by a single trained observer (AA), blinded to subject details. Image quality was graded as 0 = not analysable, 1 = fair (artefact present but images still analysable), 2 = good (artefact present but not in the region of interest), 3 = excellent. Blinding was achieved by anonymising the scans using unique study codes, performing batch analysis of all scans in a random order and allowing at least 2 weeks’ gap for the intra-observer variability analysis.

### LA volumes and EF

**Fig. 1:**
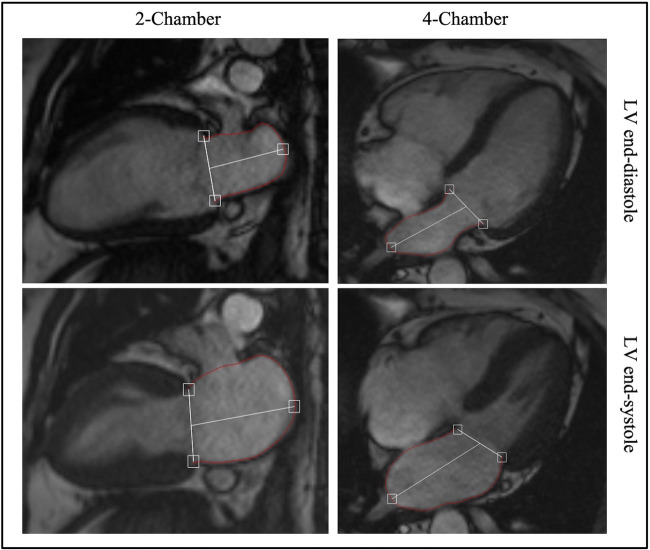
Left atrial volume quantification for emptying fraction calculation. Cine 2- and 4-chamber images illustrating contoured maximum (at left ventricular systole) and minimum (at left ventricular diastole) left atrial areas for calculating LA volume and emptying fraction using biplane area length method

LA maximal volume (LAVmax) at end-ventricular systole and LA minimal volume (LAVmin) at end-ventricular diastole were quantified using the biplane area length method [7] on 2- and 4-chamber cine images, using QMass v8.1 (Medis Suite v3.1 Medical imaging systems) (Fig. 1). LA volume pre-atrial contraction (LAVpre-A) was measured during ventricular diastole in the cine frame immediately prior to atrial contraction. LA area was automatically generated by the software after identifying the junction points of the anterior and posterior mitral annulus with the LA wall and an additional reference point on the LA roof (maximum length). Insufficient tracing was manually adjusted as required. The LA appendage and pulmonary veins were excluded from LA volumetric measurements. LAEF was calculated corresponding to the three LA phases: *reservoir function* (LA relaxation allowing the collection of venous return during LV systole), *conduit function* (passive LV filling during early diastole) and *contraction booster-pump* (active LV filling by LA contraction during late diastole [26] according to the following equations:

$$\mathrm{LA}\ \mathrm{total}\ \mathrm{EF}\ \left( reservoir\ function\right)=\left[\left(\mathrm{LAVmax}-\mathrm{LAVmin}\right)/\mathrm{LAVmax}\right]\times 100\%$$

$$\mathrm{LA}\ \mathrm{passive}\ \mathrm{EF}\ \left( conduit\ function\right)=\left[\left(\mathrm{LAVmax}-\mathrm{LAVpre}-\mathrm{A}\right)/\mathrm{LAVmax}\right]\times 100\%$$

$$\mathrm{LA}\ \mathrm{active}\ \mathrm{EF}\ \left( booster- pump\ function\right)=\left[\left(\mathrm{LAVpre}-\mathrm{A}-\mathrm{LAVmin}\right)/\mathrm{LAVpre}-\mathrm{A}\right]\times 100\%$$

### LA strain and strain rate

**Fig. 2:**
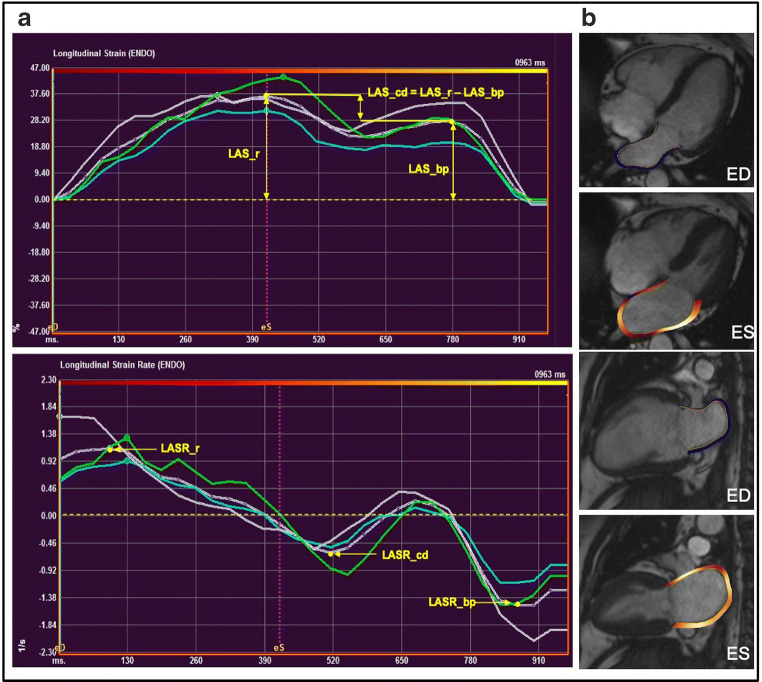
An example of left atrial strain and strain rate assessment using Feature Tracking. **a** A typical LA strain curve (upper) and a typical strain rate curve (lower), anterior wall (green), inferior wall (blue), LA roof (white) and the average (purple dotted line). **b** 4-Chamber and 2-chamber images illustrate LA contours at ventricular end-systole and end-diastole (LAS_r, LA strain at reservoir; LAS_cd, LA strain at conduit; LAS_bp, LA strain at booster-pump phase; LASR_r, LA strain rate at reservoir; LASR_cd, LA strain rate at conduit; LASR_bp, LA strain rate at booster-pump phase)

LAS and LASR were assessed with feature tracking, using QStrain v2.0 (Medis Suite v3.1, Medical imaging systems) from the 2- and 4-chamber cine images and average values calculated (Fig. 2). LA endocardial borders were traced at ventricular end-diastole and end-systole, excluding the LA appendage and pulmonary veins, and the software automatically propagated contours to the rest of the cardiac cycle. Contour adjustment was only possible on the end-ventricular diastole and end-ventricular systole phases, and was done in around 30% and 75% of the cases, respectively, to ensure the exclusion of the appendage and pulmonary veins from the LA volume. Strain and strain rate curves were obtained by identifying end-ventricular diastole as the time reference (a value of zero designated as the baseline) in line with prior recommendations [27]. LAS and LASR were also measured for the three LA phases [27]: *reservoir function* (LAS_r and LASR_r), *conduit function* (LAS_cd and LASR_cd) and *contraction booster-pump* (LAS_bp and LASR_bp). LAS_cd was calculated as: LAS_cd = LAS_r−LAS_bp.

### Reproducibility and observer variability analyses

Image analysis for test-retest reproducibility was conducted by a single observer (AA) who also performed the intra-observer variability assessment. The intra-observer assessment was performed on 10 scans that were randomly selected using an online random selection generator (a mixture of AS, T2D and healthy volunteers) for repeat analysis, performed at least 2 weeks apart. For inter-observer variability assessment, the same scans were analysed by a second blinded observer (BAM).

### Statistical analysis

Statistical tests were performed using SPSS version 26.0 software (Statistical Package for the Social Sciences). Normality was assessed using the Shapiro-Wilk test, histograms and Q-Q plots. Numerical data are expressed as mean ± standard deviation (SD) and categorical data are expressed as counts and percentages. Test-retest reproducibility and intra/inter-observer variability were assessed using the Bland-Altman method to test the limits of agreement [28], two-way mixed-effect intraclass correlations (ICC) calculated for absolute agreement and the coefficient of variance (CoV) [29] to assess the range between the mean and SD of the difference. ICC was scored as follows: excellent > 0.90, good 0.75–0.90, moderate 0.50–0.75 and poor < 0.50 [30], while CoV was scored as excellent < 10%, good 10–20%, moderate 20–30% and poor > 30%. The differences were expressed as percentages in Bland-Altman plots, calculated as: [(scan1−scan 2)/mean) × 100] [31]. For continuous variables, one-way analysis of variance (ANOVA) was used to determine significant differences across the groups, whilst unpaired *t* tests were used to assess differences between two groups. Pearson’s correlation was used to assess correlation between techniques.

## Results

Demographic data and LV parameters for the participants are shown in Table 1. The AS cohort was older, with the majority being male in the AS and haemodialysis groups. Patients with T2D had the highest BMI. Echocardiographically measured peak and mean pressure gradients were 66.1 ± 21.0 mmHg and 39.9 ± 14.5 mmHg, respectively, for the AS cohort. The average time taken to perform LA strain analysis was 9.42 ± 1.2 min with an extra 5.7 ± 0.4 min to extract the values for strain and strain rate from the curves and calculate the average. The average time to quantify LAV/LAEF using the biplane method was 5.9 ± 0.8 min. All images were analysable, and image quality was rated as good or excellent in all cases (23 (38.3%) and 37 (61.7%), respectively).

**Table 1:** Baseline demographic and left ventricle parameters using CMR

Variable	All participants (*n* = 60)	Aortic stenosis (*n* = 16)	Type 2 diabetes (*n* = 28)	Haemodialysis (*n* = 10)	Healthy volunteers (*n* = 6)
Age (years)	60.5 ± 11.1	67.3 ± 9.1	57.9 ± 10.2	57.8 ± 15.0	58.5 ± 6.9
Male (*n*, (%))	39 (65%)	13 (81.3%)	14 (50%)	8 (80%)	4 (66.7%)
BMI (kg/m^2^)	29.5 ± 5.3	27.1 ± 4.2	32.8 ± 5.2	25.0 ± 1.9	28.0 ± 2.7
BSA (m^2^)	1.97 ± 0.2	1.94 ± 0.2	2.05 ± 0.2	1.78 ± 0.2	2.00 ± 0.2
SBP (mmHg)	144.4 ± 19.6	148.1 ± 21.1	142.3 ± 16.4	142.6 ± 27.2	146.6 ± 18.0
DBP (mmHg)	82.6 ± 11.6	76.1 ± 10.5	88.0 ± 6.3	72.8 ± 14.7	91.4 ± 8.7
Heart rate (beats per minute)	71.0 ± 9.6	71.0 ± 10.9	75.4 ± 10.9	72.8 ± 11.2	64.7 ± 5.5
**Medical and drug history**					
Hypertension	38 (63.3%)	10 (62.5%)	17 (60.7%)	9 (90%)	2 (33.3%)
Diabetes	34 (56.7%)	3 (18.8%)	28 (100%)	3 (30%)	0 (0%)
CAD	5 (8.3%)	2 (12.5%)	0 (0%)	3 (30%)	0 (0%)
ACEi	11 (18.3%)	3 (18.8%)	7 (25%)	0 (0%)	1 (16.7%)
ARB	9 (15%)	2 (12.5%)	5 (17.9%)	2 (20%)	0 (0%)
Beta-Blocker	12 (20%)	7 (43.8%)	1 (3.6%)	4 (40%)	0 (0%)
Statin	35 (58.3%)	12 (75%)	15 (53.6%)	7 (70%)	1 (16.7%)
**CMR left ventricular data**					
LVEDV (ml)	149.9 ± 41.6	177.5 ± 48.8	136.1 ± 38.0	139.3 ± 21.0	158.3 ± 29.4
LVEDVI (ml/m^2^)	76.3 ± 18.5	91.7 ± 19.6	65.7 ± 13.6	79.3 ± 14.1	79.3 ± 11.0
LVEF (%)	62.8 ± 8.6	57.4 ± 5.3	68.4 ± 6.5	54.3 ± 7.2	64.9 ± 6.7
LV Mass (g)	116.4 ± 28.8	127.2 ± 35.9	116.9 ± 23.8	95.2 ± 22.0	120.3 ± 26.8
LVMI (g/m^2^)	59.0 ± 11.1	65.4 ± 14.0	56.9 ± 7.7	53.8 ± 11.5	60.0 ± 9.6
LVM/LVEDV (g/ml)	0.80 ± 0.2	0.72 ± 0.1	0.89 ± 0.2	0.68 ± 0.1	0.76 ± 0.1

Data represented as mean ± SD or number (%)

*SBP* systolic blood pressure, *DBP* diastolic blood pressure, *CAD* coronary artery disease, *ACEi* angiotensin-converting enzyme inhibitor, *ARB* angiotensin-receptor blocker, *CCB* calcium channel blocker, *LVEDVI* left ventricular end-diastolic volume index, *LVEF* left ventricular ejection fraction, *LVMi* left ventricular mass index

### Test-retest reproducibility

#### LA volumes and EF

LA volumes were highest in the AS cohort, being statistically significant compared to the T2D cohort only, whilst total LAEF was significantly lower in all patient groups compared to controls (*p* < 0.05) (Supplemental Table 1). The test-retest reproducibility of LA volumes and LAEF was good to moderate for all participants; LAVmax CoV = 19.6% ICC = 0.89, LAVmin CoV = 27.0% ICC = 0.89, LAVpre-A CoV = 22.5% ICC 0.89, and total LAEF CoV = 15.6% ICC = 0.78 (Table 2 and Supplemental Table 2).

**Table 2:** Test-retest reproducibility of LA volumes and emptying fraction using area length method for all participants (*n* = 60)

Parameters	Scan 1	Scan 2	Bias ± SD difference	BA limits of agreement	CoV(%)	ICC
LAVmax (ml)	72.1 ± 23.1	77.2 ± 25.5	− 5.1 ± 14.6	23.6, − 33.7	19.6	0.89
LAVmin (ml)	35.0 ± 14.8	36.9 ± 16.2	− 1.9 ± 9.7	17.1, 20.9	27.0	0.89
LAVpre-A (ml)	54.8 ± 19.6	59.6 ± 22.5	− 4.8 ± 12.9	20.5, − 30.0	22.5	0.89
Total LAEF (%)	51.9 ± 10.2	53.2 ± 9.1	− 1.4 ± 8.2	14.7, − 17.4	15.6	0.78

Data represented as mean ± SD

*LAV (max/min/pre-A)* left atrial volume (maximal/minimal/pre-atrial contraction), *LAEF* left atrial emptying fraction, *BA* Bland-Altman; *CoV* coefficient of variance, *ICC* intraclass correlation

#### LA strain and strain rate

The strain values for reservoir (LAS_r) and conduit (LAS_cd) phases were higher in the healthy control group compared to patient groups, reaching statistical significance when compared to haemodialysis and AS groups respectively. The booster-pump phase (LAS_bp) was highest in the AS group, being significantly higher than the T2D and haemodialysis groups (*p* = 0.02 and *p* = 0.009 respectively). The strain rate for the reservoir phase (LASR_r) was higher in healthy subjects compared to T2D and haemodialysis groups (*p* = 0.013 and *p* = 0.012 respectively) (Supplemental Table 1).

The test-retest reproducibility of LAS and LASR for the overall cohort and separate groups are shown in Table 3 and Supplemental Table 3. The reproducibility was moderate to poor for all strain and strain rate parameters. Overall, LAS and LASR corresponding to reservoir phase was the most reproducible, with the smallest CoVs (LAS_r 29.9%, LASR_r 28.9%).

**Table 3:** Test-retest reproducibility of LA strain and strain rates using Feature Tracking for all participants (*n* = 60)

Parameters	Scan 1	Scan 2	Bias ± SD difference	BA limits of agreement	CoV (%)	ICC
LAS_r (%)	28.1 ± 8.6	29.5 ± 8.4	− 1.4 ± 8.6	15.5, − 18.2	29.9	0.66
LAS_cd (%)	13.8 ± 6.3	14.0 ± 6.5	0.23 ± 5.5	10.5, − 11.0	39.5	0.78
LAS_bp (%)	14.3 ± 7.1	14.5 ± 5.6	− 0.22 ± 5.8	11.2, − 11.6	40.3	0.74
LASR_r (s^−1^)	1.0 ± 0.3	0.9 ± 0.3	0.03 ± 0.3	0.6, − 0.5	28.9	0.68
LASR_cd (s^−1^)	− 0.7 ± 0.3	− 0.6 ± 0.3	− 0.01 ± 0.2	0.5, − 0.5	35.9	0.83
LASR_bp (s^−1^)	− 1.0 ± 0.4	− 1.0 ± 0.4	− 0.05 ± 0.4	0.7, − 0.9	40.7	0.62

Data represented as mean ± SD

*LAS/SR_r* left atrial strain/strain rate at reservoir phase, *LAS/SR_cd* left atrial strain/strain rate at conduit phase, *LAS/SR_bp* left atrial strain/strain rate at booster-pump phase, *BA* Bland-Altman, *CoV* coefficient of variance, *ICC* intraclass correlation

**Fig. 3:**
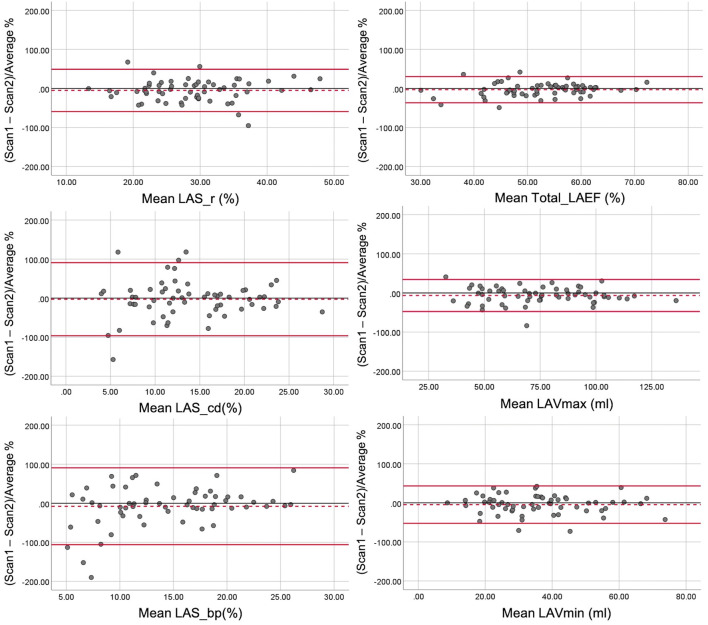
Bland-Altman plots for test-retest reproducibility of left atrial strain (left panel) and volume (right panel) parameters (LAS_r, LA strain at reservoir; LAS_cd, LA strain at conduit; LAS_bp, LA strain at booster-pump phase; LAV, left atrial volume; LAEF, left atrial emptying fraction)

Overall, the test-retest reproducibility of biplane method for assessing LA function was better than the reproducibility of strain assessment, with lower CoVs and narrower limits of agreement on Bland-Altman plots illustrated in Fig. 3.

#### Correlation

**Fig. 4:**
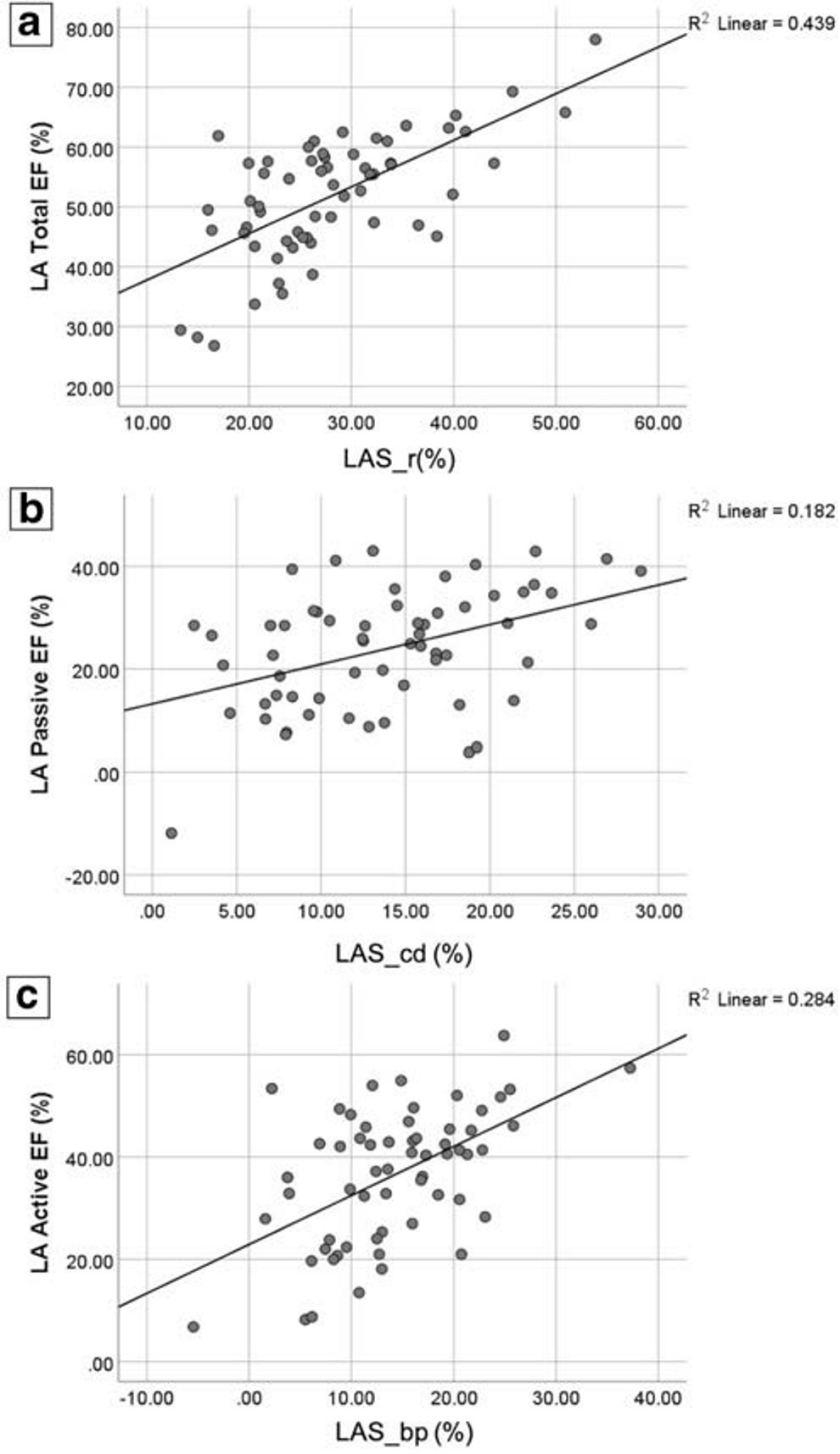
Associations of left atrial emptying fractions with left atrial strain parameters. Scatter plots showing the correlation between total LA emptying fraction (EF) and LA strain at reservoir phase (**a**), passive LAEF and LA strain at conduit phase (**b**), active LAEF and LA strain at contraction booster-pump phase (**c**) (LAS_r, LA strain at reservoir; LAS_cd, LA strain at conduit; LAS_bp, LA strain at booster-pump phase; LAEF, left atrial emptying fraction)

Figure 4 shows the correlation between LAEF at the three LA phases during the cardiac cycle and the corresponding strain parameter. There was a moderate correlation between total LAEF and LAS_r (Pearson’s correlation *r* = 0.66, *p* < 0.001). Weak but significant correlations were found between Passive LAEF *vs* LAS_cd (*r* = 0.43, *p* = 0.001) and Active LAEF *vs* LAS_bp (*r* = 0.53, *p* < 0.001).

#### Intra- and inter-observer variability

The intra- and inter-observer variability of LA strain and volumes are shown in Supplemental Tables 4 and 5. Overall, the intra- and inter-observer variability was good for all parameters except for conduit function using strain analysis. LA volumetric assessment using biplane method had better inter- and intra-observer variability compared to strain assessment.

## Discussion

To our knowledge, this is the first study to include patients and the largest cohort of subjects to have test-retest reproducibility assessment of novel LA strain parameters and routinely used LA volume parameters, using CMR. Our primary finding is that assessing the LA volumetric function by biplane method is more reproducible and quicker to analyse than the novel strain assessment technique using Feature Tracking. We found a modest correlation between the two imaging techniques.

### Test-retest reproducibility

Most studies assessing reproducibility of an imaging technique focus on inter- and intra-observer variability [32–34]. However, whilst important, observer variability does not address the multiple potential sources of variability when assessing the LA function, including image planning and acquisition, inclusion or exclusion of pulmonary veins/LA appendage from the LA volume as well as day-to-day physiological variation and filling status of the subject. Furthermore, studies assessing test-retest reproducibility of LA assessment are limited and with small sample sizes (*n* = 16–22) [35, 36] and none in clinical populations. Test-retest reproducibility of an imaging technique is fundamental for its validity and its appropriate use in longitudinal studies for monitoring changes with disease progression or in response to treatment.

We found LA volume and LAEF to be more reproducible than LA strain parameters. This has been noted previously in a study of 22 healthy subjects [35], although they used Tissue Tracking, a similar image analysis technique. Similar to our findings, LA strain corresponding to reservoir function has previously been shown to be the most reproducible strain parameter in 16 healthy subjects [36]. However, in that study, strain parameters were found to be more reproducible than volumes, contrary to our results. Their repeat scans were done on the same day, immediately following the first scan, which may have led to reduced variability in planning of the imaging, especially if the same radiographer performed both image acquisitions without blinding. The contradictory results may also be due to differences between various CMR image analysis platforms and vendor software packages, since the algorithm used to produce strain and strain rate curves differ [37]. Furthermore, especially for LA deformation analysis, the zero line for the strain curves is different across vendors [2, 27]. This is also true for LV deformation analysis where the test-retest reproducibility using tissue tracking has been found to be superior to that of feature tracking [38].

The poor test-test reproducibility of LA strain could also be related to the change LA filling states between the two scans. However, it has been shown that strain is less affected by preload than the volume assessment [39] and our date shows volumetric assessment to have better test-test reproducibility than strain.

Despite the calculation that is based on geometric assumptions [40], biplane area length has been increasingly used in clinical practice. This is due to it being a faster alternative to short-axis discs method, that does not require additional slices that increase the scan time, with more breathing instructions. It has been shown that the biplane area length method is a reliable and reproducible technique in CMR [41, 42]. This is supported by our results showing better test-retest reproducibility in comparison with LA strain analysis.

### Correlation between LA volumetric and strain parameters

We found moderate correlations between the strain and volumetric parameters corresponding to the reservoir phase of LA function, which corresponds to the main LA assessment phase in routine clinical practice (total LAEF). One other previous study has looked at the correlation between the LA volume and LA strain parameters, and found good correlation, especially for reservoir function [33].

### LA strain as a potential novel imaging parameter

It has been shown that LA deformation has an incremental role in assessing disease progression and states, since it detects LA functional impairment at early stages before changes in LA size become evident [16, 43]. LA reservoir and conduit function using CMR feature tracking has recently been shown to be abnormal in the early stages of hypertension even before LVH develops [44]. We have shown that LAS and LASR assessments are feasible by CMR feature tracking using routinely acquired SSFP sequences, as previously reported [33]. Although tissue tagging is considered to be the gold standard for LV strain analysis by CMR, its utility in quantifying LA deformation is severely limited by spatial resolution, due to the LA wall being very thin [45]. CMR feature tracking therefore offers a feasible technique to quantify LA deformation, using routinely acquired cine images. However, it has poor test-retest reproducibility and is a relatively time-consuming technique in the clinical practice setting. Therefore, further developments are required before this technique can be recommended for use in routine clinical practice or as an outcome measure in clinical research studies.

### Limitations

This study has some limitations. The number of participants in each group is small and heterogeneous. However, test-retest reproducibility studies are rare, and the overall number is the largest to date reporting LAS reproducibility. The purpose was to assess the reproducibility of the imaging technique rather than assess differences between cohorts. We used a single software package for the image analysis and the result may not apply to other available software. Importantly, both strain and volume assessment were analysed using the same software. Patients were studied at both 1.5 Tesla and 3 Tesla scanners and hence some of the variability in our results may be due to inherent differences in reproducibility between field strengths. However, the two scans for each participant were performed on the same scanner with the same field strength. Blood pressure was not recorded during LA cine acquisition during the scans, and a baseline blood pressure was only recorded for the first CMR. However, medication remained unchanged between the two scans.

## Conclusions

The test-retest reproducibility of LAS and LASR by CMR utilising Feature Tracking is moderate to poor across disease states, whereas LA volume and emptying fraction are more reproducible on CMR. Further development of CMR LA strain quantification methods is needed before this can be recommended in for clinical use.

## Electronic Supplementary Material

ESM 1 (DOCX 29.4 kb)

## Abbreviations

AS: Aortic stenosis
CoV: Coefficient of variance
LA: Left atrium
LAEF: Left atrial emptying fraction
LAS/SR_bp: Left atrial strain/strain rate at booster-pump phase
LAS/SR_cd: Left atrial strain/strain rate at conduit phase
LAS/SR_r: Left atrial strain/strain rate at reservoir phase
LAV (max/min/pre-A): Left atrial volume (maximal/minimal/pre-atrial contraction)
T2D: Type 2 diabetes
