# Supplementary material for: Reproducibility of left atrial function using cardiac magnetic resonance imaging
Source: Eur Radiol. 2020 Oct 30;31(5):2788–97. doi: 10.1007/s00330-020-07399-z (PMC8043954; doi:10.1007/s00330-020-07399-z)
Supplement: Supplementary file 1 — (DOCX 29.4 kb) [file 330_2020_7399_MOESM1_ESM.docx]

**Online only Supplementary Tables**

**Supplemental Table 1:** Comparative analysis between the study groups.

| **Parameters** | **Aortic Stenosis (n=16)** | **Type 2 Diabetes (n=28)** | **Haemodialysis (n=10)** | **Healthy volunteers (n=6)** | **Statistical significance difference (P-value)** | | | | | | |
| --- | --- | --- | --- | --- | --- | --- | --- | --- | --- | --- | --- |
|  |  |  |  |  | **P-value**  ***(overall)***  ***ANOVA*** | **AS vs T2D** | **AS vs HD** | **AS vs HV** | **T2D vs HD** | **T2D vs HV** | **HD vs HV** |
| **LA volumes and emptying fraction using Area-length method** | | | | |  | | | | | | |
| LAVmax (ml) | 83.0 ±23.1 | 67.6 ± 21.2 | 69.1 ± 27.0 | 69.2 ± 20.8 | 0.18 | **0.04*** | 0.20 | 0.21 | 0.87 | 0.87 | 0.99 |
| LAVmin (ml) | 42.4 ± 16.4 | 31.7 ± 12.5 | 36.5 ± 17.3 | 28.6 ± 10.2 | 0.08 | **0.03*** | 0.40 | **0.03*** | 0.44 | 0.53 | 0.27 |
| LAVpre-A (ml) | 67.2 ± 22.0 | 50.1 ± 16.1 | 51.3 ± 19.6 | 49.9 ± 18.1 | **0.03*** | **0.01*** | 0.07 | 0.09 | 0.87 | 0.98 | 0.89 |
| Total LAEF (%) | 48.9 ± 8.9 | 53.6 ± 9.9 | 47.3 ± 12.7 | 59.2 ± 4.0 | 0.06 | 0.12 | 0.73 | **0.001*** | 0.18 | **0.04*** | **0.02*** |
| **LA strain and strain rates using Feature Tracking** | | | | |  | | | | | | |
| LAS_r (%) | 29.0 ± 6.9 | 28.2 ± 10.1 | 23.5 ± 7.2 | 33.1 ± 4.3 | 0.16 | 0.75 | 0.06 | 0.12 | 0.12 | 0.07 | **0.005*** |
| LAS_cd (%) | 11.2 ± 6.0 | 15.1 ± 5.6 | 11.6 ± 6.4 | 18.6 ± 6.5 | **0.03*** | **0.04*** | 0.88 | **0.04*** | 0.16 | 0.26 | 0.06 |
| LAS_bp (%) | 17.9 ± 5.3 | 13.1 ± 8.1 | 11.9 ± 5.0 | 14.5 ± 6.8 | 0.11 | **0.02*** | **0.009*** | 0.32 | 0.60 | 0.66 | 0.43 |
| LASR_r (s^-1^) | 1.0 ± 0.3 | 0.9 ± 0.3 | 0.9 ± 0.2 | 1.1 ± 0.1 | 0.25 | 0.39 | 0.28 | 0.12 | 0.74 | **0.013*** | **0.012*** |
| LASR_cd (s^-1^) | -0.5 ± 0.2 | -0.7 ± 0.3 | -0.7 ± 0.4 | -0.8 ± 0.4 | 0.06 | **0.007*** | 0.19 | 0.08 | 0.78 | 0.53 | 0.48 |
| LASR_bp (s^-1^) | -1.1 ±0.4 | -1.0 ± 0.4 | -0.9 ± 0.4 | -0.9 ± 0.5 | 0.31 | 0.42 | 0.09 | 0.19 | 0.23 | 0.42 | 0.82 |

Data represented as mean ± SD

Abbreviations: LAV(max/min/pre-A) = Left atrial volume (maximal/minimal/pre-atrial contraction), LAEF= left atrial emptying fraction, LAS_r = LA strain at reservoir, LAS_cd= LA strain at conduit, LAS_bp= LA strain at booster-pump phase, LASR_r = LA strain rate at reservoir, LASR_cd= LA strain rate at conduit, LASR_bp= LA strain rate at booster-pump phase, AS= Aortic stenosis, T2D= Type 2 diabetes, HD= Haemodialysis, HV= healthy volunteers.

* Indicate statistically significant difference (p <0.05).

**Supplemental Table 2:** Test-retest reproducibility of LA volumes and emptying fraction using Area-length method across diseases.

| **Parameters** | **Scan 1** | **Scan 2** | **Bias ±SD Difference** | **BA limits of agreement** | **CoV(%)** | **ICC** |
| --- | --- | --- | --- | --- | --- | --- |
| **Aortic Stenosis Patients (n=16)** | | | | | | |
| LAVmax (ml) | 83.0 ±23.1 | 86.3± 27.3 | -3.3 ± 17.4 | 30.8, -37.4 | 20.6 | 0.87 |
| LAVmin (ml) | 42.4 ± 16.4 | 44.4 ± 17.4 | -2.0 ±14.5 | 26.4, -30.4 | 33.4 | 0.78 |
| LAVpre-A (ml) | 67.2 ± 22.0 | 70.1 ± 23.8 | -2.9 ± 14.9 | 26.3, -32.2 | 21.8 | 0.88 |
| Total LAEF (%) | 48.9 ± 8.9 | 49.4 ± 5.8 | -0.5 ± 8.4 | 16.0, -16.9 | 17.1 | 0.56 |
| **Diabetic patients (n=28)** | | | | | | |
| LAVmax (ml) | 67.6 ± 21.2 | 70.7 ± 25.3 | -3.2 ± 10.6 | 17.6, -23.9 | 15.3 | 0.94 |
| LAVmin (ml) | 31.7 ± 12.5 | 32.1 ± 14.5 | -0.4 ± 5.1 | 9.6, -10.5 | 16.1 | 0.96 |
| LAVpre-A (ml) | 50.1 ± 16.1 | 53.2 ± 22.1 | -3.0 ± 11.9 | 20.2, -26.3 | 23.0 | 0.90 |
| Total LAEF (%) | 53.6 ± 9.9 | 55.5 ± 9.0 | -1.9 ± 8.5 | 14.8, -18.5 | 15.6 | 0.75 |
| **Haemodialysis patients (n=10)** | | | | | | |
| LAVmax (ml) | 69.1 ± 27.0 | 80.6 ± 23.2 | -11.4 ± 19.8 | 27.4, -50.2 | 26.5 | 0.78 |
| LAVmin (ml) | 36.5 ± 17.3 | 41.3 ± 17.4 | -4.8 ± 10.6 | 16.0, -25.7 | 27.3 | 0.89 |
| LAVpre-A (ml) | 51.3 ± 19.6 | 63.4 ± 19.5 | -12.1 ± 12.8 | 12.9, -37.1 | 22.3 | 0.80 |
| Total LAEF (%) | 47.3 ± 12.7 | 50.1 ± 10.8 | -2.8 ± 4.7 | 6.3, -11.9 | 9.6 | 0.95 |
| **Healthy volunteers (n=6)** | | | | | | |
| LAVmax (ml) | 69.2 ± 20.8 | 77.5 ± 21.3 | -8.3 ± 13.3 | 17.7, -34.3 | 18.1 | 0.87 |
| LAVmin (ml) | 28.6 ± 10.2 | 32.5 ± 12.1 | -3.9 ± 10.1 | 15.9, -23.6 | 33.0 | 0.75 |
| LAVpre-A (ml) | 49.9 ± 18.1 | 55.6 ± 18.2 | -5.7 ± 9.9 | 13.8, -25.1 | 18.8 | 0.91 |
| Total LAEF (%) | 59.2 ± 4.0 | 58.2 ± 10.3 | 1.0 ± 11.6 | 23.8, -21.8 | 19.8 | 0.32 |

Data represented as mean ± SD

Abbreviations as in supplemental table 1 and BA= Bland-Altman CoV= coefficient of variance, ICC= intraclass correlation.

**Supplemental Table 3:** Test-retest reproducibility of LA strain and strain rates using Feature Tracking across diseases.

| **Parameters** | **Scan 1** | **Scan 2** | **Bias ±SD Difference** | **BA limits of agreement** | **CoV(%)** | **ICC** |
| --- | --- | --- | --- | --- | --- | --- |
| **Aortic Stenosis Patients (n=16)** | | | | | | |
| LAS_r (%) | 29.0 ± 6.9 | 28.0± 6.5 | 1.04 ± 6.8 | 14.5, -12.4 | 24.1 | 0.66 |
| LAS_cd (%) | 11.2 ± 6.0 | 10.6 ± 5.9 | 0.53 ± 6.6 | 13.5, -12.4 | 60.4 | 0.57 |
| LAS_bp (%) | 17.9 ± 5.3 | 17.3 ± 5.3 | 0.51 ± 5.4 | 11.0, -9.98 | 30.4 | 0.67 |
| LASR_r (s^-1^) | 1.0 ± 0.3 | 0.9 ± 0.3 | 0.09 ± 0.3 | 0.60, -0.43 | 27.8 | 0.71 |
| LASR_cd (s^-1^) | -0.5 ± 0.2 | -0.5 ± 0.3 | 0.00 ± 0.2 | 0.35, -0.35 | 36.7 | 0.86 |
| LASR_bp (s^-1^) | -1.1 ±0.4 | -1.0 ± 0.3 | -0.13 ± 0.4 | 0.7, -1.0 | 40.3 | 0.49 |
| **Diabetic patients (n=28)** | | | | | | |
| LAS_r (%) | 28.2 ± 10.1 | 30.8 ± 9.9 | -2.7 ± 10.8 | 18.3, -23.7 | 36.3 | 0.59 |
| LAS_cd (%) | 15.1 ± 5.6 | 14.5 ± 5.4 | 0.6 ± 5.3 | 10.8, -9.7 | 35.5 | 0.71 |
| LAS_bp (%) | 131 ± 8.1 | 14.4 ± 5.4 | -1.3 ± 7.1 | 12.5, -15.1 | 51.4 | 0.65 |
| LASR_r (s^-1^) | 0.9 ± 0.3 | 0.9 ± 0.3 | 0.02 ± 0.3 | 0.51, -0.47 | 27.9 | 0.76 |
| LASR_cd (s^-1^) | -0.7 ± 0.3 | -0.7 ± 0.3 | -0.05 ± 0.2 | 0.43, -0.52 | 24.6 | 0.78 |
| LASR_bp (s^-1^) | -1.0 ± 0.4 | -1.1 ± 0.4 | 0.02 ± 0.4 | 0.8, -0.8 | 38.6 | 0.68 |
| **Haemodialysis patients (n=10)** | | | | | | |
| LAS_r (%) | 23.5 ± 7.2 | 25.4 ± 7.0 | -2.0 ± 3.8 | 5.4, -9.4 | 15.5 | 0.91 |
| LAS_cd (%) | 11.6 ± 6.4 | 14.1 ± 7.2 | -2.6 ± 3.4 | 4.0, -9.2 | 26.2 | 0.91 |
| LAS_bp (%) | 11.9 ± 5.0 | 11.3 ± 4.7 | -0.6 ± 3.3 | 7.1, -5.9 | 28.5 | 0.88 |
| LASR_r (s^-1^) | 0.9 ± 0.2 | 1.0 ± 0.3 | -0.1 ± 0.3 | 0.44, -0.63 | 29.2 | 0.63 |
| LASR_cd (s^-1^) | -0.7 ± 0.4 | -0.7 ± 0.3 | 0.02 ± 0.3 | 0.53, -0.49 | 37.5 | 0.86 |
| LASR_bp (s^-1^) | -0.85 ± 0.4 | -0.78 ± 0.4 | -0.07 ± 0.5 | 0.82, -0.97 | 55.9 | 0.28 |
| **Healthy volunteers (n=6)** | | | | | | |
| LAS_r (%) | 33.1 ± 4.3 | 33.7 ± 5.0 | -0.60 ± 7.5 | 14.1, -15.3 | 22.5 | 0.84 |
| LAS_cd (%) | 18.6 ± 6.5 | 20.6 ± 7.9 | -2.0 ± 6.1 | 9.9, -14.0 | 31.1 | 0.79 |
| LAS_bp (%) | 14.5 ± 6.8 | 13.1 ± 6.4 | 1.4 ± 3.6 | 8.5, -5.7 | 26.2 | 0.92 |
| LASR_r (s^-1^) | 1.1 ± 0.1 | 1.0 ± 0.3 | 0.1 ± 0.4 | 0.8, -0.6 | 33.7 | 0.89 |
| LASR_cd (s^-1^) | -0.8 ± 0.4 | -0.9 ± 0.3 | 0.03 ± 0.3 | 0.7, -0.6 | 39.4 | 0.7 |
| LASR_bp (s^-1^) | -0.9 ± 0.5 | -0.8 ± 0.4 | -0.1 ± 0.2 | 0.3, -0.6 | 28.3 | 0.89 |

Data represented as mean ± SD

Abbreviations as in supplemental table 1 and BA= Bland-Altman CoV= coefficient of variance, ICC= intraclass correlation.

**Supplemental Table 4:** Inter-observer variability of LA assessment (n=10).

| **Parameters** | **Scan 1**  **(mean±SD)** | **Scan 2**  **(mean±SD)** | **Bias ± SD Difference**  **(scan1-scan2)** | **BA Limits of agreement** | **CoV (%)** | **ICC** |
| --- | --- | --- | --- | --- | --- | --- |
| **LA volumes/LAEF (Area-length method)** | | | | | | |
| LAVmax (ml) | 71.3±20.3 | 66.7±22.9 | 4.7±10.6 | 25.5, -16.1 | 15.4 | 0.93 |
| LAVmin (ml) | 33.1±11.3 | 29.5±11.3 | 3.6±6.2 | 15.7, -8.4 | 19.7 | 0.90 |
| LAVpre-A (ml) | 52.3±14.4 | 48.5±18.2 | 3.8±8.8 | 21.0, -13.4 | 17.4 | 0.92 |
| Total LAEF (%) | 54.2±6.8 | 56.4±3.6 | -2.2±4.1 | 5.9, -10.3 | 7.5 | 0.81 |
| **Strain/Strain rate (Feature Tracking)** | | | | | | |
| LAS_r (%) | 26.6±4.0 | 29.1±3.3 | -2.55±5.5 | 8.1, -13.2 | 19.6 | 0.17 |
| LAS_cd (%) | 11.2±4.7 | 12.1±6.3 | -0.87±5.0 | 9.0, -10.7 | 43.2 | 0.76 |
| LAS_bp (%) | 15.4±4.9 | 17.1±5.0 | -1.7±2.2 | 2.6, -6.0 | 13.4 | 0.66 |
| LASR_r (s^-1^) | 0.89±0.15 | 0.83±0.2 | 0.06±0.2 | 0.3, -0.2 | 13.6 | 0.55 |
| LASR_cd (s^-1^) | -0.5±0.3 | -0.4±0.3 | -0.04±0.3 | 0.5, -0.5 | 56.1 | 0.85 |
| LASR_bp (s^-1^) | -1.0±0.3 | -1.1±0.2 | 0.06±0.2 | 0.5, -0.42 | 22.7 | 0.75 |

Data represented as mean ± SD

Abbreviations as in supplemental table 1 and BA= Bland-Altman CoV= coefficient of variance, ICC= intraclass correlation.

**Supplemental Table 5:** Intra-observer variability of LA assessment on cardiac MRI (n=10).

| **Parameters** | **Scan 1**  **(mean±SD)** | **Scan 2**  **(mean±SD)** | **Bias ± SD Difference**  **(scan1-scan2)** | **BA Limits of agreement** | **CoV (%)** | **ICC** |
| --- | --- | --- | --- | --- | --- | --- |
| **LA volumes/LAEF (Area-length method)** | | | | | | |
| LAVmax (ml) | 71.3±20.3 | 70.3±20.1 | 1.04±5.7 | 12.2, -10.1 | 8.06 | 0.98 |
| LAVmin (ml) | 33.1±11.3 | 32.5±10.9 | 0.57±4.0 | 8.4, -7.3 | 12.2 | 0.97 |
| LAVpre-A (ml) | 52.7±14.4 | 52.3±15.7 | 0.02±4.2 | 8.2, -8.1 | 7.93 | 0.98 |
| Total LAEF (%) | 54.2±6.8 | 53.3±7.2 | 0.90±3.4 | 8.7, -6.9 | 7.43 | 0.92 |
| **Strain/Strain rate (Feature Tracking)** | | | | | | |
| LAS_r (%) | 27.3±6.1 | 26.6±4.0 | 0.72±4.3 | 9.2, -7.8 | 16.1 | 0.80 |
| LAS_cd (%) | 11.9±6.5 | 11.2±4.7 | 0.73±3.9 | 8.3, -6.9 | 33.6 | 0.88 |
| LAS_bp (%) | 15.4±5.8 | 14.4±4.9 | -0.01±4.4 | 8.6, -8.6 | 28.5 | 0.82 |
| LASR_r (s^-1^) | 0.9±0.3 | 0.9±0.1 | 0.03±0.1 | 0.3, -0.2 | 15.1 | 0.89 |
| LASR_cd (s^-1^) | -0.5±0.3 | -0.5±0.3 | -0.04±0.1 | 0.2, -0.3 | 24.0 | 0.96 |
| LASR_bp (s^-1^) | -1.0±0.3 | -1.0±0.3 | -0.01±0.2 | 0.2, -0.2 | 8.94 | 0.97 |

Data represented as mean ± SD

Abbreviations as in supplemental table 1 and BA= Bland-Altman CoV= coefficient of variance, ICC= intraclass correlation.
